# Supplementary material for: Global distribution of particle phase state in atmospheric secondary organic aerosols
Source: Nat Commun. 2017 Apr 21;8:15002. doi: 10.1038/ncomms15002 (PMC5413943; doi:10.1038/ncomms15002)
Supplement: Supplementary Information — Supplementary Figures, Supplementary Tables and Supplementary References [file ncomms15002-s1.pdf]

Supplementary Table 1. Molar mass (*M*), O:C and T_g for SOA products in volatility bins.

Volatility (μg m^{-3})	Anthropogenic SOA			Biogenic SOA		
	M (g mol^{-1})	O:C	T_g (K)	*M* (g mol^{-1})	O:C	T_g (K)
1	362	0.6	326	293	0.4	298
10	249	0.4	276	203	0.24	234
100	227	0.3	256	185	0.14	211
1000	205	0.25	236	167	0.1	192

Supplementary Table 2. Molar mass distributions for sensitivity studies of T_g prediction.

Volatility (μg m^{-3})	Anthropogenic SOA		Biogenic SOA	
	High case	Low case	High case	Low case
1	434	290	352	234
10	299	199	244	162
100	272	182	222	148
1000	246	164	200	134

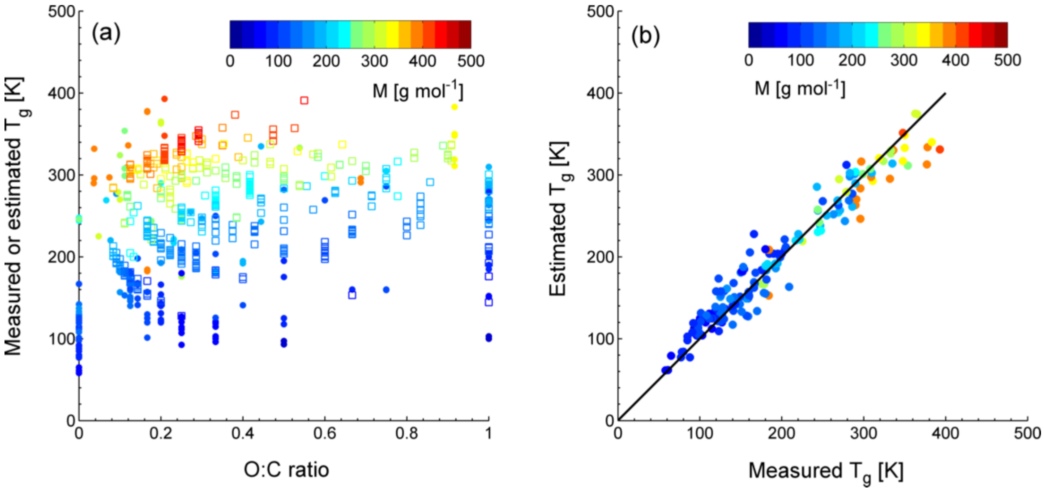

Supplementary Figure 1. Characteristic relations between molecular O:C ratio and glass transition temperature of organic compounds. (a) Measured (circles) and estimated (squares) glass transition temperature (T_g) of organic compounds as a function of O:C ratio. Organic compounds with measured T_g are from Koop et al. (2011)[1] and Dette et al. (2014)[2]. Those with estimated T_g are 654 SOA components from Shiraiwa et al. (2014)[3]. (b) Comparison of measured and estimated T_g for 179 organic compounds[1,2]. The markers are color-coded by molar mass. The correlation coefficient is 0.97, demonstrating that the estimation method of T_g using EPI and the Boyer-Kauzmann rule is adequate.

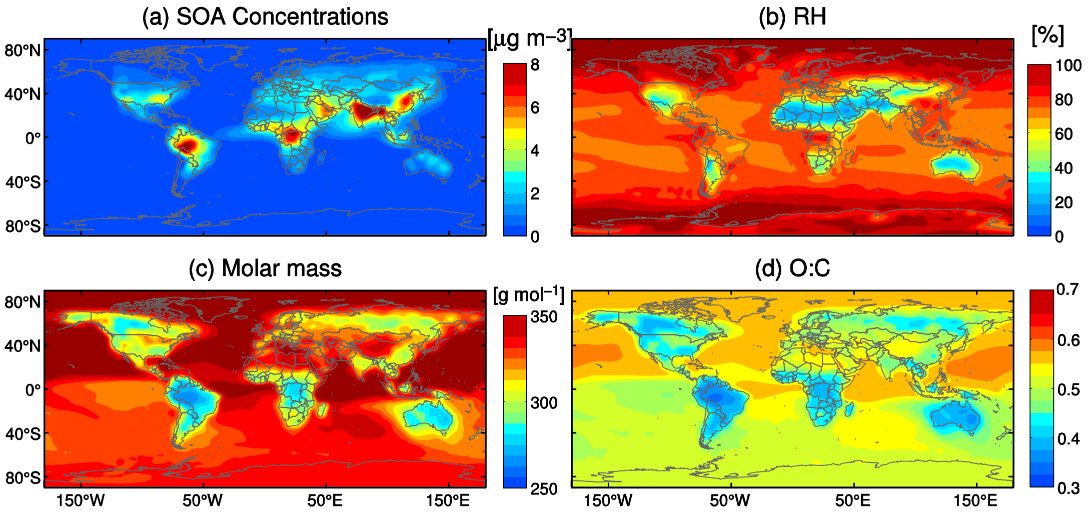

Supplementary Figure 2. Global modeling of SOA. Modeled annual averages of (a) SOA concentrations, (b) relative humidity, (c) molar mass and (d) O:C at the surface during the years 2005-2009.

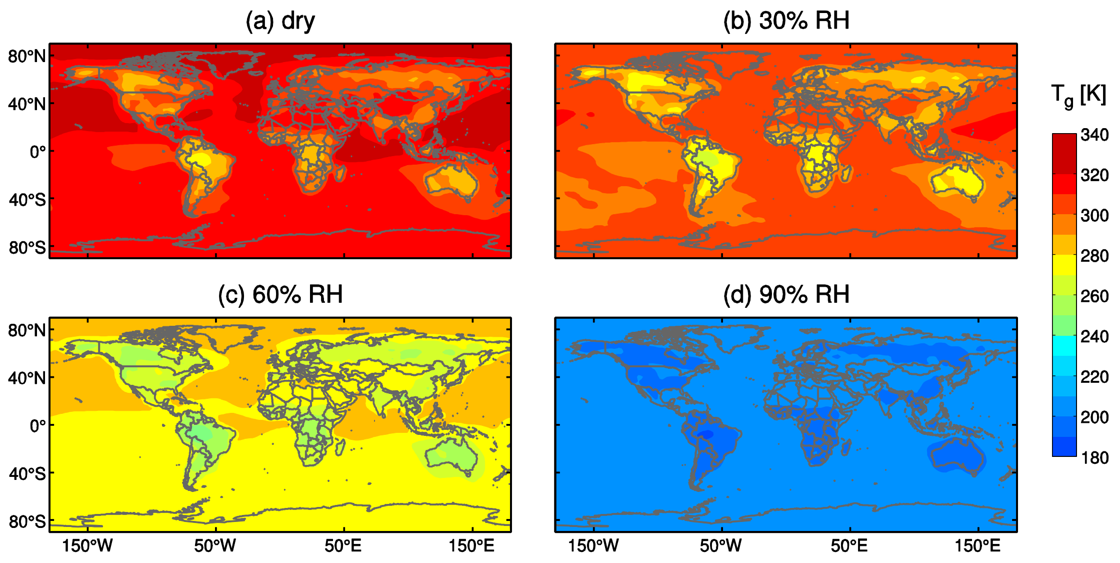

Supplementary Figure 3. Modeled annual averages of T_g of SOA particles. (a) Dry condition, (b) 30% RH, (c) 60% RH, and (d) 90% RH at the surface during the years 2005-2009.

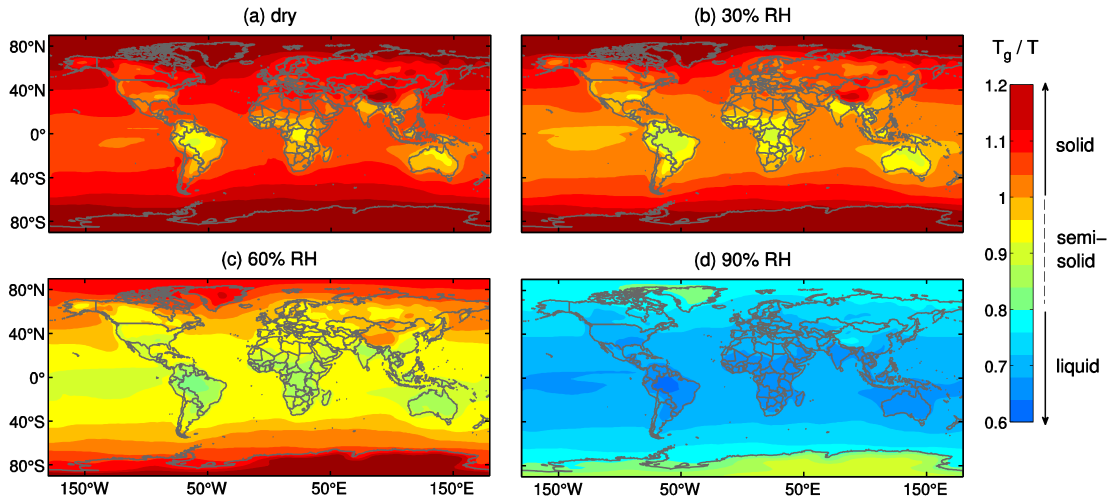

Supplementary Figure 4. Modeled annual averages of the inverse ambient temperature (1/*T*) scaled by the glass transition temperature (T_g) of SOA (T_g/T). (a) Dry condition, (b) 30% RH, (c) 60% RH, and (d) 90% RH at the surface during the years 2005-2009.

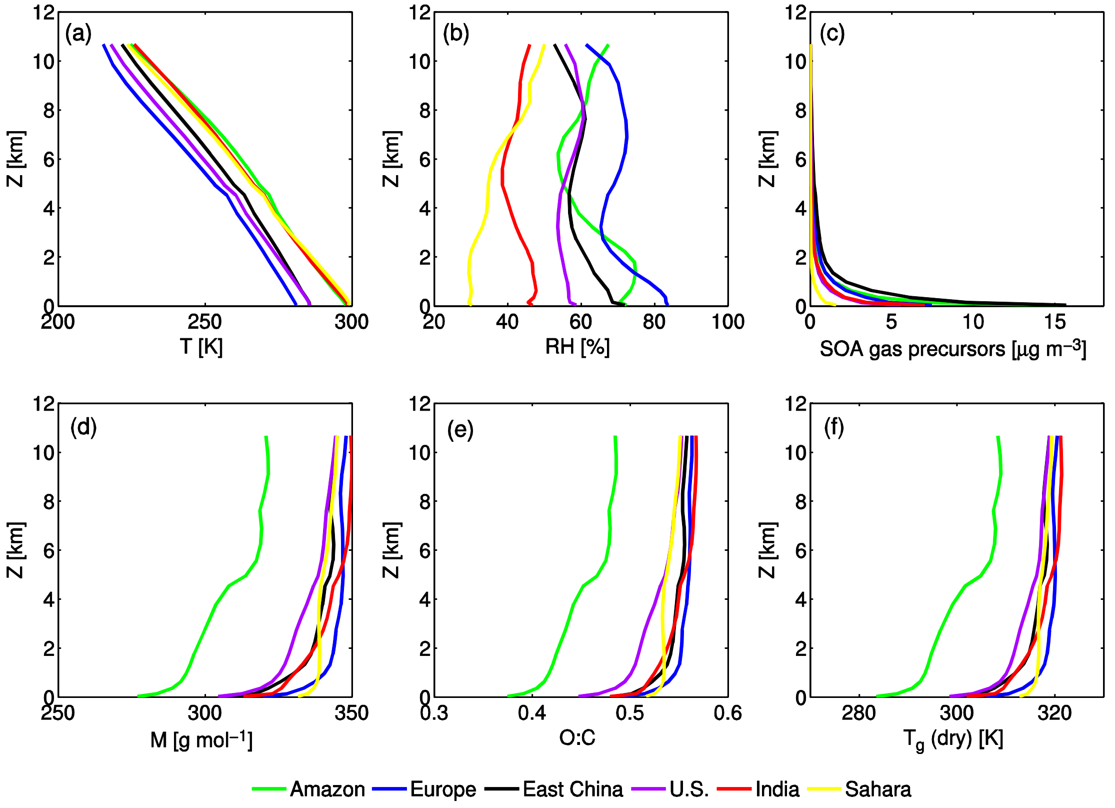

Supplementary Figure 5. Modeled mean vertical profiles. (a) ambient temperature, (b) relative humidity, (c) concentrations of SOA gas precursors, (d) molar mass, (e) O:C, and (f) T_g of dry SOA. The simulation grids covered by the Amazon basin, Europe, East China, U.S., India and Sahara are shown in Fig. 2(a).

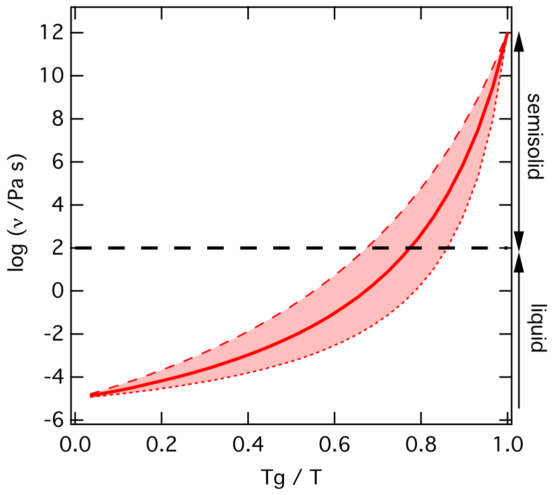

Supplementary Figure 6. The Angell plot of viscosity vs. T_g / T. The lines represent different fragility (*D*) of *D* = 10 (the solid line) as the base case for this study as well as *D* = 5 (the dotted line) and *D* = 20 (the dashed line) representing the possible range for SOA. The black dashed line at viscosity of 10^2 Pa s indicates the threshold of liquid and semisolid states.

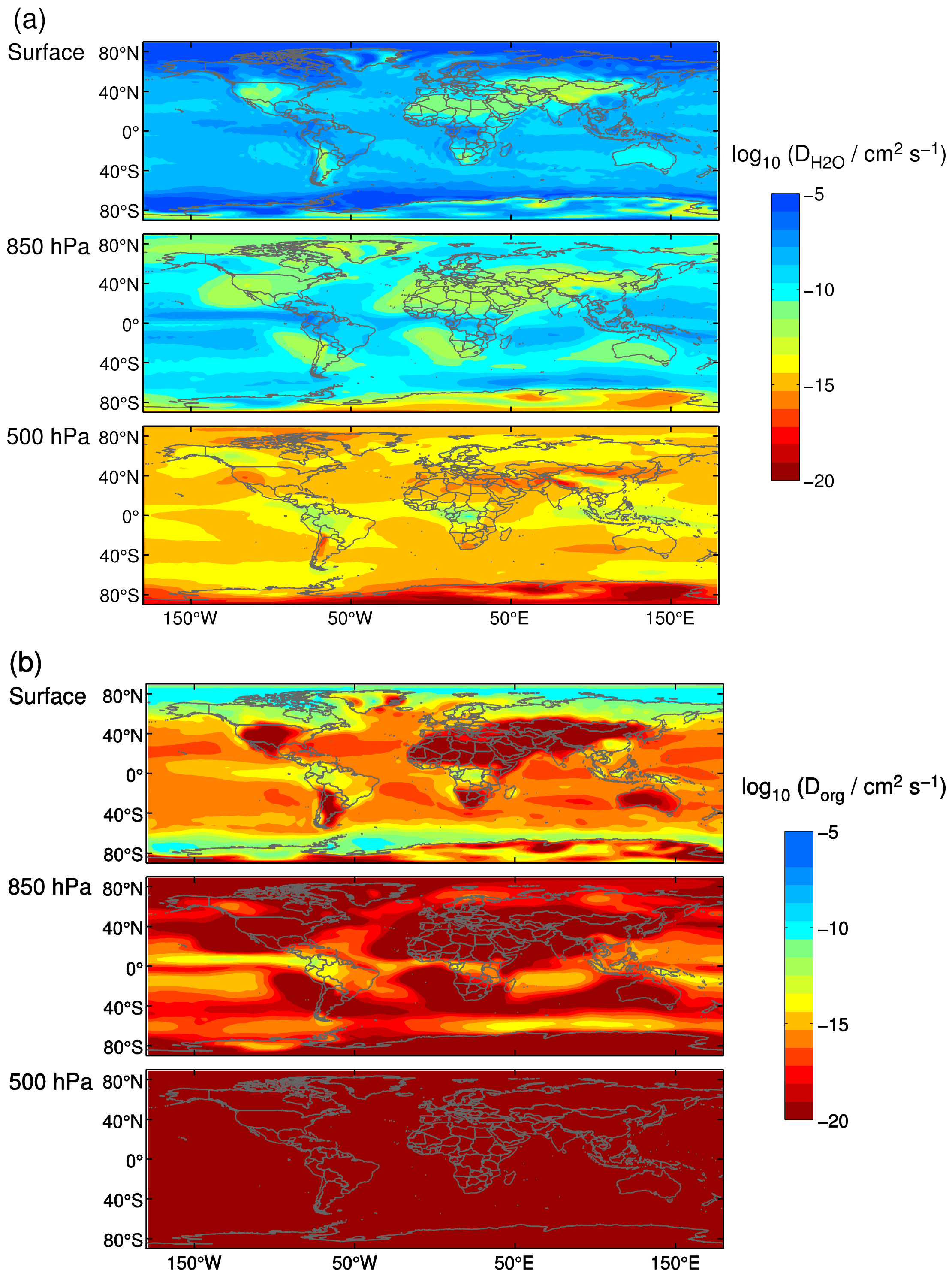

Supplementary Figure 7. Global maps of estimated bulk diffusivities. Bulk diffusivities ($cm^2\ s^{-1}$) of (a) water and (b) organic molecules in SOA particles at the surface, 850 hPa, and 500 hPa.

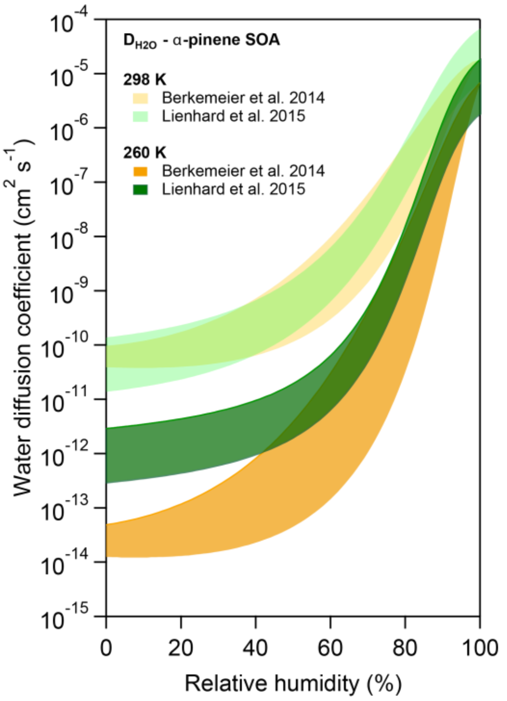

Supplementary Figure 8. Water diffusivity in α-pinene SOA. Comparison of water diffusion coefficients in α-pinene SOA (O:C ratio = 0.54) obtained from water uptake experiments in an electrodynamic balance[4] (green shaded areas) with values obtained with the semi-empirical estimation method presented in Berkemeier et al. (2014)[5] (orange shaded areas) at 260 K and 298 K. Water diffusivities were calculated with O:C = 0.5, $T_{g,SOA}$ = 278.5±7 K, κ_{org}= 0.12±0.02, k_{GT}=1.5±1, ρ_{org} = 1.4 g cm^{-3}.

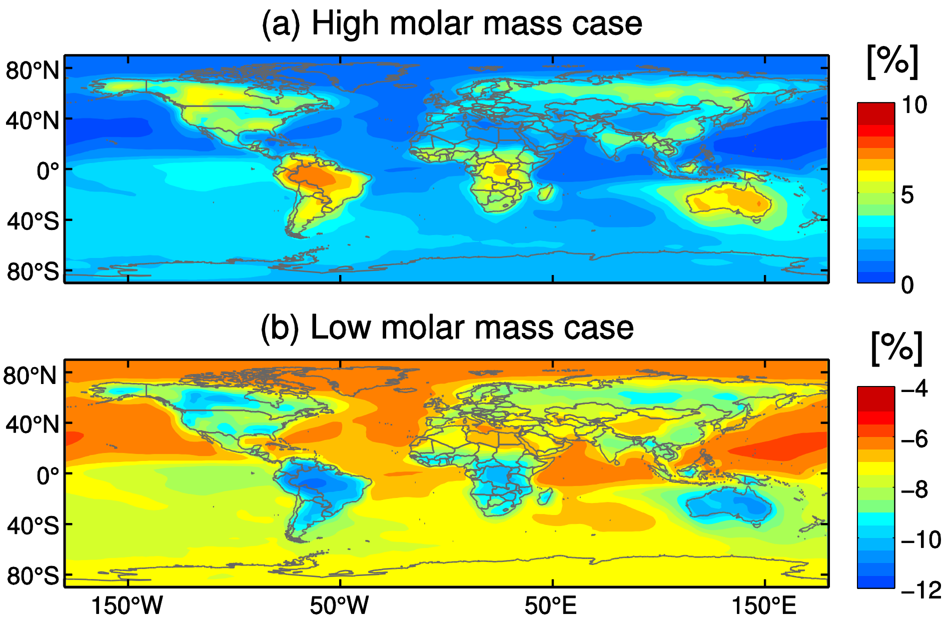

Supplementary Figure 9. Sensitivity of molar mass on T_g. Modeled annual mean differences of T_g for SOA (without water) between a base case (Table 1) and cases with (a) high and (b) low molar mass values assigned in volatility bins (Supplementary Table 2), respectively.

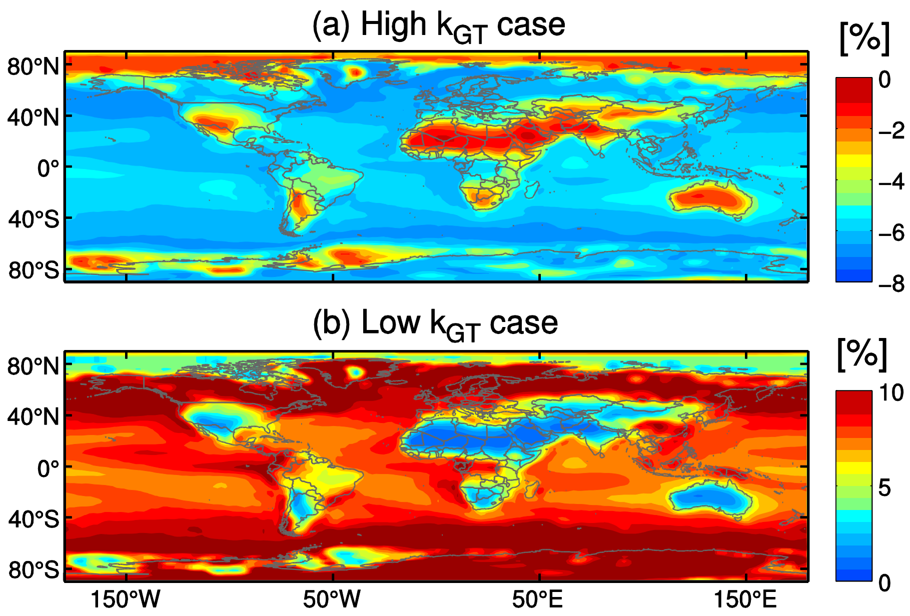

Supplementary Figure 10. Sensitivity of k_{GT} on T_g. Modeled annual mean differences of T_g for SOA between a base case with the Gordon-Taylor constant of k_{GT} equaling to 2.5 and cases with (a) a higher k_{GT} of 3.5 and (b) a lower k_{GT} of 1.5, respectively.

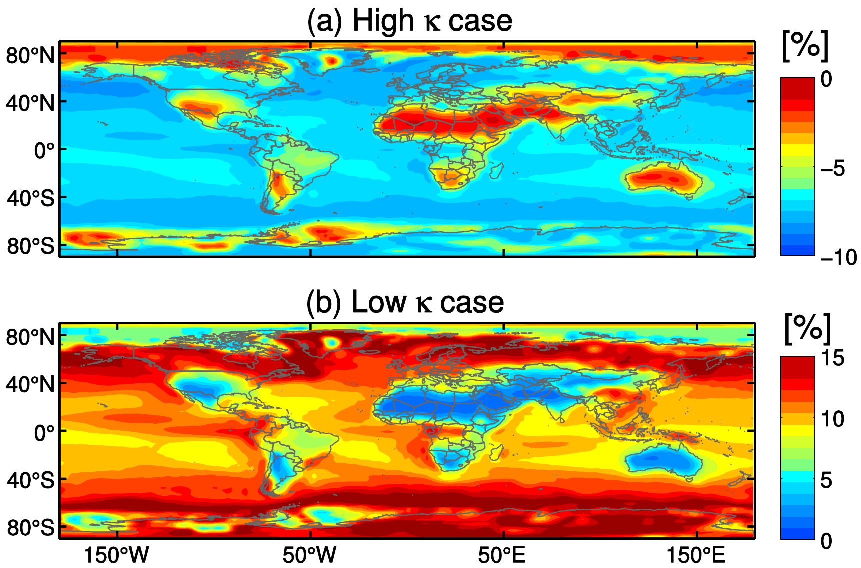

Supplementary Figure 11. Sensitivity of κ on T_g. Modeled annual mean differences of T_g for SOA between a base case with the hygroscopicity parameter of κ equaling to 0.1 and cases with (a) a higher κ of 0.15 and (b) a lower κ of 0.05, respectively.

Supplementary references.

1. Koop, T., Bookhold, J., Shiraiwa, M. & Pöschl, U. Glass transition and phase state of organic compounds: dependency on molecular properties and implications for secondary organic aerosols in the atmosphere. *Phys. Chem. Chem. Phys.* **13**, 19238-19255 (2011).
2. Dette, H. P., Qi, M., Schröder, D. C., Godt, A. & Koop, T. Glass-forming properties of 3-Methylbutane-1,2,3-tricarboxylic acid and its mixtures with water and pinonic acid. *J. Phys. Chem. A* **118**, 7024-7033 (2014).
3. Shiraiwa, M., Berkemeier, T., Schilling-Fahnestock, K. A., Seinfeld, J. H. & Pöschl, U. Molecular corridors and kinetic regimes in the multiphase chemical evolution of secondary organic aerosol. *Atmos. Chem. Phys.* **14**, 8323-8341 (2014).
4. Lienhard, D. M. *et al.* Viscous organic aerosol particles in the upper troposphere: diffusivity-controlled water uptake and ice nucleation? *Atmos. Chem. Phys.* **15**, 13599-13613 (2015).
5. Berkemeier, T., Shiraiwa, M., Pöschl, U. & Koop, T. Competition between water uptake and ice nucleation by glassy organic aerosol particles. *Atmos. Chem. Phys.* **14**, 12513-12531 (2014).
